# Enhancing capacitance behaviour of CoOOH nanostructures using transition metal dopants by ambient oxidation

**DOI:** 10.1038/srep20704

**Published:** 2016-02-08

**Authors:** Yanhui Chen, Junfeng Zhou, Pierce Maguire, Robert O’Connell, Wolfgang Schmitt, Yonghe Li, Zhengguang Yan, Yuefei Zhang, Hongzhou Zhang

**Affiliations:** 1 Institute of Microstructure and Property of Advanced Materials, Beijing University of Technology, Beijing, 100124, China; 2 School of Physics and Centre for Research on Adaptive Nanostructures and Nanodevices (CRANN), Trinity College Dublin, Dublin 2, Republic of Ireland; 3 School of Chemistry and Centre for Research on Adaptive Nanostructures and Nanodevices (CRANN), Trinity College Dublin, Dublin 2, Republic of Ireland

## Abstract

Cobalt hydrate and doped binary Co_0.9_M_0.1_OOH (M = Ni, Mn, Fe) nanorings of 100–300 nm were fabricated in solution through a facile ambient oxidation method. A transformation from Co_0.9_Ni_0.1_(OH)_2_ nanodiscs to hollow Co_0.9_Ni_0.1_OOH nanorings was observed with prolonged reaction time. Core-shell nanodiscs have elemental segregation with a Co(OH)_2_ core and Ni(OH)_2_ shell. Co_0.9_Ni_0.1_OOH nanorings displayed a higher electrochemical capacitance than Mn and Fe doped nanorings materials or materials with disc-like geometries.

Hollow inorganic nanomaterials have attracted intense research interest due to low mass density, high porosity and increased surface area^1^,^2^,^3^. These characteristics make them good candidate materials for high surface area devices such as lithium-ion batteries and gas sensors^4^,^5^,^6^,^7^,^8^. Nanorings are one of the hollow structures which have been successfully fabricated and exhibit extraordinary properties due to their ring-like morphology^9^,^10^. Divalent metal oxides or their hydrates’ nanorings are the most intensively studied hollow materials due to their high surface-to-volume ratio and special magnetic properties^10^,^11^,^12^. CoOOH is an active material used in conjunction with nickel hydroxide in Ni–H cells. Cobalt oxide is coated on the positive electrodes of rechargeable alkaline nickel batteries to achieve high electrochemical efficiency^13^. In addition, CoOOH is non-stoichiometric and the Co is at a higher oxidation state (+3) than in Co_3_O_4_ (the oxidation states are +4,+3,+2 and the average is +8/3)^14^,^15^. Usually the synthesis process for cobalt/ion hydrates contains a microwave irradiation/ultrasonic chiseling pre-treatment and a high temperature (140 ^o^C–230 ^o^C) post-hydrothermal reaction for few hours^16^,^17^,^18^ with low yield^19^. Cobalt hydroxide nanorings have been reported with polycrystalline structure after 10 h in a sealed autoclave at 180 ^o^C^20^. Hexagonal Co_3_O_4_ nanorings have also been produced from precursor Co(OH)_2_ nanodiscs at 160 °C for 10 h with diameters of a few micrometres^12^. Co-Sn-O systems have also been reported using a hydrothermal method at high temperature via the Kirkendall effect through the diffusion of pores^21^. However, other doping such as Mn, or Fe into the Co(OH)_2_ is still not reported. A facile room temperature method is needed to prepare doped cobalt hydroxide nanorings with high yield.

Here, we will give detailed preparation and analysis on Co_0.9_Ni_0.1_OOH nanorings via ambient oxidation. Typical images of Co_0.9_Ni_0.1_OOH products under different reaction times will be discussed, revealing the shape transformation and growth mechanism. Electrochemical properties of CoOOH materials doped with different elements will also be presented.

## Results and Discussion

**Figure 1:**
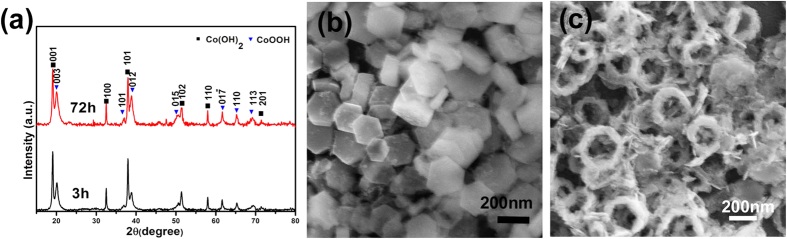
(**a**) XRD patterns of products after reaction for 3 h and 72 h. Typical SEM image of the synthesized materials for different reaction times for (**b**) 3 h and (**c**) 72 h.

Figure 1 shows the XRD profiles of the products reacted for 3 h (black) and for 72 h (red). The main diffraction peaks in the black spectrum can be attributed to the hexagonal trigonal β-Co(OH)_2_ structure (JCPDS file No. 30-0443, space group: P3-m1 (166); lattice constants: a = 3.2 Å, c = 4.6 Å, α = 90°, β = 120°). While weak peaks in the spectrum of 3 h products can be attributed to the β-CoOOH structure (JCPDS file No. 73–1213, space group: Fd-3m (227); lattice constants: a = 4.7 Å, α = 35.5°). After reaction for 72 h, the peaks of CoOOH were observed to be increased compared to those from Co(OH)_2_. This indicates that oxidation continues to occur during this prolonged reaction. Representative SEM images of nanodiscs and nanorings are provided in Fig. 1b,c which displays an outer diameter of 200–300 nm and mean diameter of 230 nm. Statistical SEM results indicate that the yield of nanorings is more than 95% (quantity) in products reacted for 72 h. It can be seen that the surface of the nanorings is rough and composed of loose particles while the inner rings are close to hexagonal shape.

**Figure 2:**
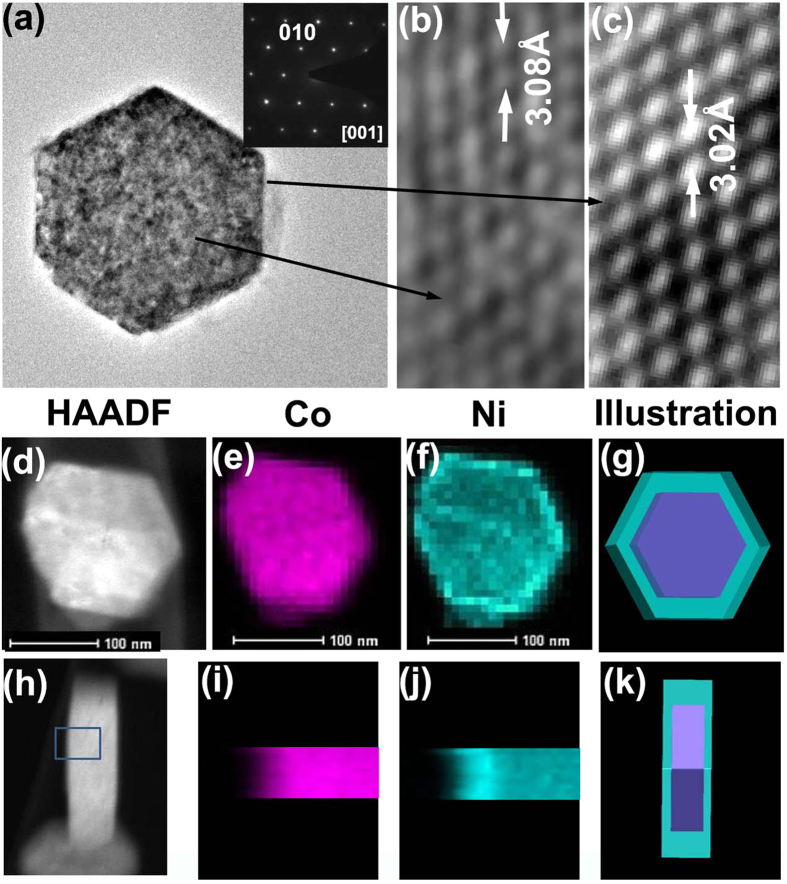
(**a**) TEM image of a typical nanodisc and its corresponding diffraction pattern. (**b,c**) HRTEM image on the inner and border of the disc. (**d–g**) EDX mapping of one top-view nanodisc and its Co and Ni mapping and elemental distribution illustration model. (**h–k**) EDX mapping of one side-view nanodisc and its Co and Ni mapping and its elemental distribution illustration model.

Figure 2 gives typical microstructures of products prepared in 20 ^o^C for 3 h. A typical TEM image in Fig. 1a of a 220 nm outer diameter nanodisc shows that it has uniform thickness. The electron diffraction pattern inset of Fig. 2a indicates its hcp structure with lattice parameters of a = b = 3.2 Å which corresponds to β-Co(OH)_2_. High resolution transmission elecctron microscope (HRTEM) images (Fig. 2b,c) from the border and inner space of the nanodisc indicate their single crystal structure. A lattice contraction of about 2% in the <010> direction of the nanodics can be detected, a change from 3.08 Å to 3.02 Å from average measurement results. This contraction can be attributed to the nanomaterials’ border effect or structural variation from Co(OH)_2_ (lattic parameter a is 3.18 Å) to Ni(OH)_2_ (lattic parameter a is 3.11 Å), which has a contraction from about 1.6% to 2.5% in bulk material. The lattice contraction can nearly be omitted in the nanorings, while 1–3 nm amorphous layers can be detected in the outer border of most rings. A further study on the elemental distributions on the room temperature nanodiscs is shown in Fig. 2d–k. It can be seen that the nickel is preferentially distributed in a border range of about 20–30 nm, while the cobalt has a lower concentration in the border compared to the centre. The mean concentration of Ni in the border is about 27.5% while it is 6.9% in the center. A schematic illustration of the elemental distribution of the nanodiscs can be observed in the last part of Fig. 2g–k with a Co(OH)_2_ core (purplish red color) wraped by a Ni(OH)_2_ shell (azure color).

**Figure 3:**
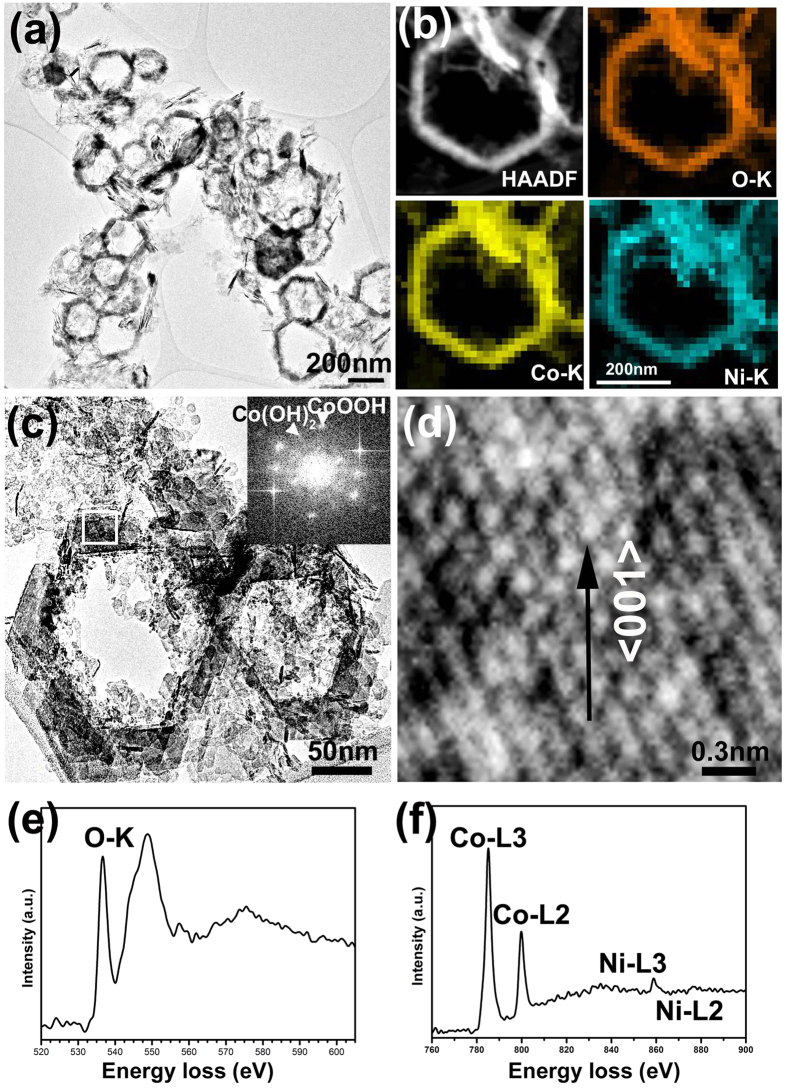
(**a**) TEM image of hexagonal Ni doped CoOOH nanorings; (**b**) HAADF STEM image and its corresponding EDX mapping of O, Co and Ni. (**c**) TEM image and FFT of part of the nanorings and (**d**) its corresponding lattice image; (**e**) EELS of O-K edge and (**f**) Co-L and Ni-L edges.

Figure 3 shows the morphologies, elemental distribution and valence state of nanorings provided from the following techniques: transmission electron microscopy (TEM), energy dispersive X-ray spectroscopy (EDX) and electron energy loss spectroscopy (EELS). In a typical low magnification TEM image in Fig. 3a, tens of rings are shown with diameters from 200–300 nm. Most of the nanorings maintain the hexagonal inner and outer border without any angle variation. The quantitative EDX results show that the ratio of Co/Ni is 87.7/12.3, which contains slightly more Ni than the starting Co/Ni ratio (9/1). A typical EDX map of one nanoring in Fig. 3b shows that the distributions of O, Co and Ni are uniform. An enlarged TEM image in Fig. 3c gives the detailed morphology of the nanorings and shows that the wall thickness of the nanorings is 30–50  nm. Although SEM observations of these nanoparticles suggest a single crystal nanoring, HRTEM results indicate an orientation related multicrystalline structure. The ring-like structure is not comprised of perfect single crystals. The inner edge of the hexagonal border is made of small nanoparticles aggregated continuously or quasi-continuously as in Fig. 3c. A thin porous structure can be seen in the centre of some ring-like structures. A HRTEM image in Fig. 3d indicates that the particles composing the hexagonal skeleton contain a single crystal structure with {001} facets of hcp CoOOH as a surface plane. Though the fast Fourier transform (FFT) of the HRTEM in the inset of Fig. 3d gives two sets of the diffraction patterns, the strong spots (indexed by the arrowhead) indicate that the main phase of the product is CoOOH, while it contains traces of the Co(OH)_2_ phase. The pre-peak in the EELS spectrum in Fig. 3e of the O edge in 535 eV indicates the existence of OH^−^ bonds^22^. Since L_3_ and L_2_ lines in Co-L edge correspond to transitions from 2p^3/2^ to 3d^3/2^3d^5/2^ and from 2p½ to 3d^3/2^ respectively, the integration of the L_3_/L_2_ ratio can provide the ratio of Co^3+^ inside it^23^. The L_3_/L_2_ ratio in the Co-L edge in our rings after calculation is about 2.2 which indicates that the products contain large quantities of trivalent cobalt. From the EELS results, we can conclude that these nanorings are mainly made up of CoOOH.

**Figure 4:**
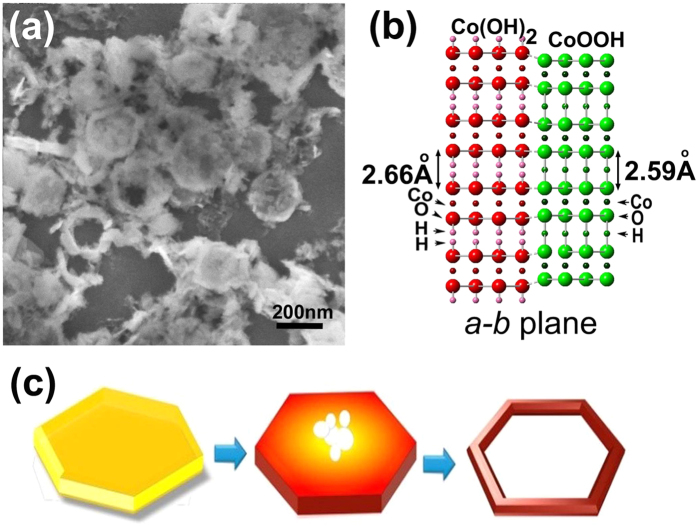
(**a**) SEM image of typical intermediate produts reacted for 24 h. (**b**) Atomic model of boundary between Co(OH)_2_ and CoOOH, in which intervals between layers contract by the loss of hydrogen atoms. (**c**) The shape evolution is illustrated schematically.

Figure 4 provides an SEM image of an intermediate state reacted for 24 h and a schematic illustration of shape evolution. In an intermediate state after reaction for 24 h as shown in Fig. 4a, some ring-like structures are formed while most of them maintain a bowl-like structure with a rough surface. The roughness of the surface can be explained by the atomic model in Fig. 4b. In Co(OH)_2_ (right part in Fig. 4b) the distance between Co-O layers is 2.66 Å, while it is 2.59 Å in CoOOH (left part in Fig. 4b) due to the loss of H atoms and an electron in the same time. In comparison of the chemical composition of Co(OH)_2_ and CoOOH, there is a H-atom removed, which in fact is referring to the removing a proton (H^+^ion) and an electron as well and there forms no H(0) species. Therefore we say the losing of H atom just in the symbolic meaning to discuss the two atomic models. More surface area in the border is bared to the oxygen-filled environment than in the inner part, resulting in enhanced oxidation of Co(OH)_2_ to CoOOH there while the inner part maintains its structure. After reaction for 72 h, ring-like structures form as shown in Fig. 1c. It indicates the formation of nanorings from the disks which first become porous and finally reach the ring-like structures.

The formation mechanism is illustrated in Fig. 4c. In the beginning, cobalt hydrate precipitates to form a disc-like core and this can be tested by EDX results on samples reacted for short times as shown in Figure S5 and Table S1 in the supplementary information. As time progresses, the loose layered borders and surfaces of β-Co(OH)_2_ begin to lose some of their H atoms and an electron due to oxidation. The outer border tends to oxidize to a contracted CoOOH structure since more areas there are exposed to the oxygen. Then a Co(OH)_2_/CoOOH boundary will be formed. Inner Co(OH)_2_ layers adjacent to the outer CoOOH will then transform/diffuse to CoOOH structure by losing H atoms. Pores will then form in the centre, eventually creating a hollow ring-like structure.

**Figure 5:**
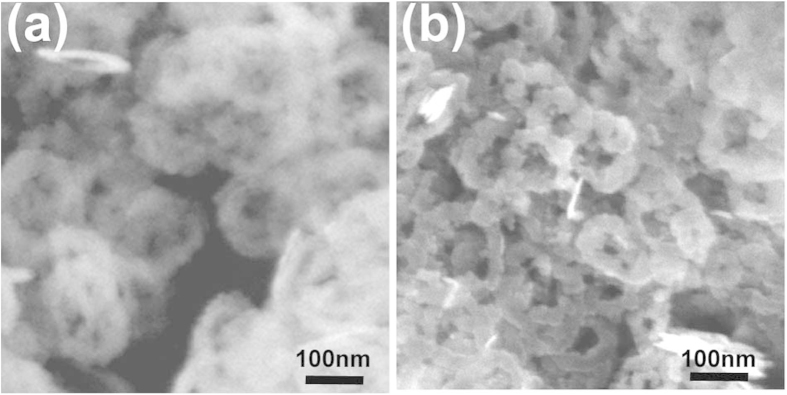
SEM image of typical doped nanorings (**a**) Co_0.9_Mn_0.1_OOH and (**b**) Co_0.9_Fe_0.1_OOH.

The lowest pH value needed for precipitation can be calculated using the following


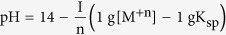


where n is the valence of the metal, M represents activity of ions of the metal and K_sp_ is the solubility product constant. In a dilute solution, the activity of ions (also known as effective concentration) can be expressed as 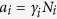 , where 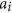, 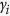 and 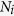 represent of activity, activity coefficient and concentration of the *ith* ion, respectively. This means that the activity 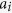 is monotonically dependent on the nominal concentration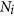. Because Co^2+^ and Ni^2+^ have the same valence value, their activities in a dilute solution are very close^24^. It is reasonable to correlate these thermodynamic quantities with the nominal concentration in this case. Calculation results indicate that if the concentration of Co^2+^ is 0.018 M, the pH value needed is 7.53. Similarly, pH values for a concentration of 0.002 M Ni, Mn and Fe are 7.93, 8.87, and 7.85 respectively. This suggests that the Co(OH)_2_ precipitate firstly forms disc-like structures due to its intrinsic hcp structure and provides borders and surfaces to which Ni/Mn/Fe(OH)_2_ can then adhere. That is the reason that no Nickel can be detected in the disc-like structures formed on short timescales while its concentration was found to have increased to 12–3% in ring-like structures (Figure S5 and Table S1). As predicted in the calculation above, Mn^2+^ and Fe^2+^ are possible doping metals with a higher pH value for precipitation needed than Co^2+^. Typical morphologies and chemical properties of Co_0.9_Mn_0.1_OOH and Co_0.9_Fe_0.1_OOH nanorings are shown in Fig. 5 and supplementary files (Figure S6-S14). The diameter of Co_0.9_Mn_0.1_OOH nanorings is about 150 nm with an average cobalt atomic composition of 91%of metal content. However, the diameter of the Co_0.9_Fe_0.1_OOH is about 100 nm with an average cobalt atomic concentration of 89%.

**Figure 6:**
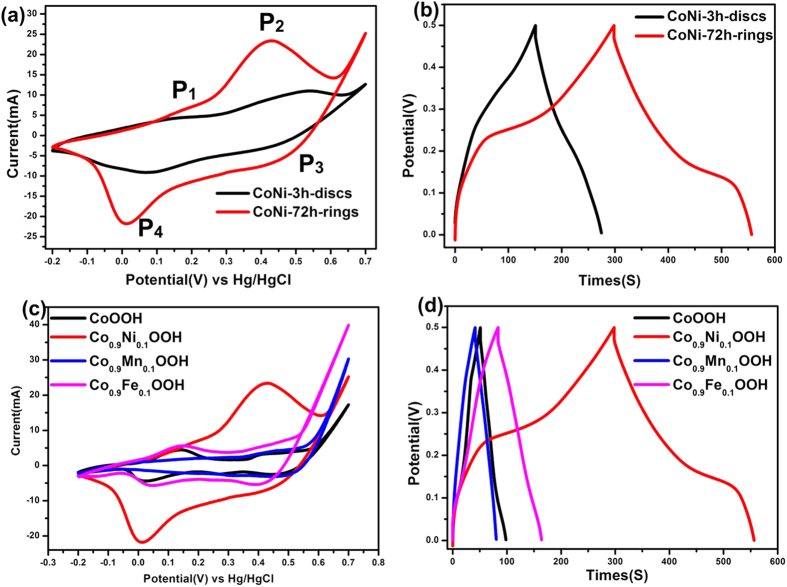
(**a**) CV curves of Co_0.9_Ni_0.1_OOH nanodiscs and nanorings at a sweep rate of 5 mVs^−1^. (**b**) Galvanostatic charge/discharge curves of Co_0.9_Ni_0.1_OOH nanodiscs and nanorings at discharge current of 1 A g^−1^. (**c**) CV curves of M doped Co_0.9_M_0.1_OOH (M = Ni, Mn, Fe) nanorings at a sweep rate of 5 mV s^−1^. (SCE). (**d**) Galvanostatic charge/discharge curves of M doped Co_0.9_M_0.1_OOH (M = Ni,Mn,Fe) nanorings at discharge current of 1 A g^−1^.

The typical cyclic voltammograms of the Co_0.9_Ni_0.1_OOH nanodiscs (black lines) and nanorings were acquired at a sweep rate of 5 mV s^−1^ with a potential window of −0.2 to 0.7 V vs a standard calomel electrode (SCE) and are shown in Fig. 6a and The four peaks of the C-V curve in Fig. 6a can be attribute to transformation of Co^2+^→Co^3+^, Co^3+^→Co^4+^, Co^4+^→Co^3+^ and Co^3+^→Co^2+^^25^. There is a distinct increase in the redox peak of the nanorings compared to that of the nanodiscs. The strong redox peak in the nanorings indicates that the capacitance characteristics are mainly governed by Faradaic reactions, which are very distinct from the rectangular shape of the electric double layer. The charge/discharge curves at current densities of 1 A g^−1^ in Fig. 6b show that after the shape changes to nanorings, its capacitance increased from 211.5 to 439.7 F g^−1^. The comparison of M doped (M = Ni, Mn, Fe) nanorings in Fig. 6c,d indicates that Ni doped CoOOH nanorings exhibit better capacitance performance than Mn and Fe doped CoOOH nanorings. The enhanced capacitance can be attribute to the porosity of the surface of the nanorings. We must point out that for pure CoOOH, the nanoring morphology enhances the capacitance but the enhancement is not as significant as the Ni doped ones^25^,^26^. This means that both the nanoring morphology and Ni doping can enhance the capacitance.

## Conclusions

In conclusion, cobalt hydrate and doped Co_0.9_M_0.1_OOH (M = Ni, Mn, Fe) nanorings were successfully fabricated by shape evolution from Co_0.9_M_0.1_(OH)_2_ nanodiscs to Co_0.9_M_0.1_OOH nanorings via ambient oxidation in room temperature. The formation and evolution process of the Co_0.9_Ni_0.1_OOH system were illustrated. Ring-like structures formed due to the contraction in the surface of the nanodiscs during the oxidation process. Co_0.9_Ni_0.1_OOH nanorings displayed a higher capacitance than its disc-like materials and other systems. Nickel doped CoOOH nanorings displayed better capacitance performance than Mn and Fe doped CoOOH nanorings.

## Methods

### Synthesis of M-doped CoOOH

For the syntheses procedure, Ni doped systems were described as an example, while synthesis with different dopants (Mn, Fe and pure CoOOH materials) use similar methods. In a typical synthesis, CoCl_2_·6H_2_O and NiCl_2_·6H_2_O were dissolved in deionized water with a ratio of 9:1. A small amount of N_2_H_4_·H_2_O was added to the solutions. During the reaction, NaOH solution was added to tune the pH value to about 13.7. This solution was continually stirred for 3–72 h in room temperature (17–20 ^o^C) while exposed to the air. A pink brown precipitate in the beginning and then a brown precipitate was collected on the bottom of the bottle. The precipitates were then washed and dried for further characterisation.

### Characterisation

An X-ray diffractometer (XRD) on a BRUKER-D8 ADVANCE diffractometer was used while employing a graphite monochromatized Cu K_α_ radiation flux at a scanning rate of 0.02° s^−1^ in the 2θ range of 1090°. A Carl Zeiss Ultra scanning electron microscope (SEM) was used to image the morphologies of the Co-Ni hydrate nanoparticles. The SEM was able to perform energy dispersive X-ray spectroscopy (EDX) on the sample. A 300 kV FEI Titan transmission electron microscope (TEM) was also used for imaging and was capable of both electron energy loss spectroscopy (EELS) and EDX. In all cases a wide selection of individual oxide nanoparticles were examined to determine their average structure and morphology.

### Electrode preparation and electrochemical tests

Electrochemical measurements were carried out in a 5 M aqueous KOH in a half-cell setup configuration at room temperature. Platinum wire and a standard calomel electrode (SCE) served as counter and reference electrodes, respectively. The working electrode was prepared by casting a paste containing Co_0.9_M_0.1_OOH nanomaterials (8 mg), carbon black, and poly(vinylidenefluoride) (PVDF) in a weight ratio of 80:10:10 onto a 1 cm × 1 cm Ni foam (2 mm thick, 100 ppi, 95% porosity, purchased from Bitaxiang Co. Ltd., Kunshan, China). The electrodes were calendared and degassed in vacuum at 80 °C for 12 h. The resulting electrode was pressed at 5 MPa to form about 150 μm thick with 1 cm*1 cm area sheet for electrochemical tests. Cyclic voltammetry (CV) and charge/discharge experiments were obtained on a CHI 660D electrochemistry workstation. CV curves were recorded at a sweep rate of 5 mV s^−1^ with a potential window of –0.2 to 0.7 V vs SCE and cycling performance was investigated at a current density of 1 A g ^−1^.

## Additional Information

**How to cite this article**: Chen, Y. *et al.* Enhancing capacitance behaviour of CoOOH nanostructures using transition metal dopants by ambient oxidation. *Sci. Rep.* **6**, 20704; doi: 10.1038/srep20704 (2016).

## Supplementary Material

Supplementary Information

## References

[b1] Caruso F., Caruso R. A. & Möhwald H. Hierarchically Ordered Oxides. Science 282, 1111 (1998).9804547

[b2] Liu D. *et al.* Co_3_O_4_ nanocages with highly exposed {110} facets for high-performance lithium storage. Sci. Rep. 3, 2543 (2013). 23995848 10.1038/srep02543 PMC3759043

[b3] Wang X. *et al.* Cobalt (ii,iii) oxide hollow structures: fabrication, properties and applications. J. Mater. Chem. 22, 23310 (2012).

[b4] Li W. Y., Xu L. N. & Chen J. Co_3_O_4_ nanomaterials in Lithium-ion batteries and gas sensors. Adv. Fun. Mater. 15, 851 (2005).

[b5] Dames P. *et al.* Targeted delivery of magnetic aerosol droplets to the lung. Nat. Nanotechnol. 2, 495 (2007). 18654347 10.1038/nnano.2007.217

[b6] Shim H. S., Shinde V. R., Kim H. J., Sung Y. E. & Kim W. B. Porous cobalt oxide thin films from low temperature solution phase synthesis for electrochromic electrode. Thin Solid Films 516, 8573 (2008).

[b7] Kartachova O. *et al.* Evolution of the electrochemical capacitance of transition metal oxynitrides with time: the effect of ageing and passivation. J. Mater. Chem. A 2, 12940 (2014).

[b8] Li Y. *et al.* Unveiling the dynamic capacitive storage mechanism of Co_3_O_4_@NiCo_2_O_4_ hybrid nanoelectrodes for supercapacitor applications. Electrochimica Acta, 145, 177 (2014).

[b9] Wu C. *et al.* Synthesis and Lithium storage properties of Co_3_O_4_ nanosheet-assembled multishelled hollow spheres. Adv. Fun. Mater. 20, 3666 (2010).

[b10] Ren S. *et al.* Facile one-pot strategy synthesis of ultrathin α-Co(OH)_2_ nanosheets towards high-performance electrochemical capacitors. Mater. Lett. 80, 23 (2012).

[b11] Xing Z. H., Wang S. S. & Xu A. W. Dipole-directed assembly of Fe_3_O_4_ nanoparticles into nanorings via oriented attachment. CrystEngComm. 16, 1482 (2014).

[b12] Dong Q., Kumada N., Yonesaki Y., Takei T. & Kinomura N. Cobalt oxide (Co_3_O_4_) nanorings prepared from hexagonal β-Co(OH)_2_ nanosheets. Mater. Res. Bull. 46, 1156 (2011).

[b13] Hu W. K., Gao X. P., Geng M. M., Gong Z. X. & Noreus D. Synthesis of CoOOH Nanorods and Application as Coating Materials of Nickel Hydroxide for High Temperature Ni−MH Cells. J. Phys. Chem. B 109, 5392 (2005). 16851567 10.1021/jp044514m

[b14] Jansson J., Palmqvist A. E. C. & Fridell E. On the catalytic activity of Co_3_O_4_ in low-temperature CO oxidation. J. Catal. 211, 387 (2002).

[b15] Grillo F., Natile M. M. & Glisenti A. Low-temperature oxidation of carbon monoxide: the influence of water and oxygen on the reactivity of a Co_3_O_4_ powder surface. Appl. Phy. B 48, 267 (2004).

[b16] Gotić M., Dražić G. & Musić S. Hydrothermal synthesis of α-Fe_2_O_3_ nanorings with the help of divalent metal cations, Mn^2+^, Cu^2+^, Zn^2+^ and Ni^2+^. J. Mol. Struct. 993, 167 (2011).

[b17] Tao B., Zhang Q., Liu Z. & Geng B. Cooperative effect of pH value and anions on single-crystalline hexagonal and circular α-Fe_2_O_3_ nanorings. Mater. Chem. Phys. 136, 604 (2012).

[b18] Lv B., Xu Y., Wu D. & Sun Y. Single-crystal α-Fe_2_O_3_ hexagonal nanorings: stepwise influence of different anionic ligands (F- and SCN- anions). Chem. Comm. 47, 967 (2011). 21072432 10.1039/c0cc03632c

[b19] Zhou D., Su X., Boese M., Wang R. & Zhang H. Ni(OH)_2_@Co(OH)_2_ hollow nanohexagons: Controllable synthesis, facet-selected competitive growth and capacitance property. Nano Energy 5, 52 (2014).

[b20] Chen Q., Wang N. & Guo L. Surfactant-free wet chemical synthesis of Co(OH)_2_ nanodisks and nanorings. Res. Chem. Intermed. 37, 421 (2011).

[b21] Peng C., Gao L. & Yang S. Synthesis and magnetic properties of Co-Sn-O nanorings, Chemical Communications. Chem. Comm. 42, 4372 (2007). 17957290 10.1039/b708803e

[b22] Wirth R. Water in minerals detectable by electron energy-loss spectroscopy EELS. Phys. Chem. Miner. 24, 561 (1997).

[b23] Wang Z. L., Yin J. S. & Jiang Y. D. EELS analysis of cation valence states and oxygen vacancies in magnetic oxides, Micron. Micron 31, 571 (2000). 10831303 10.1016/s0968-4328(99)00139-0

[b24] Atkins P. & Paula J. Physical chemistry 8th edn, (ed. Atkins P. *et al.*) 158–165 (New York, 2006).

[b25] Wang M., Ren W., Zhao Y.& Cui H. Synthesis of nanostructured CoOOH film with high electrochemical performance for application in supercapacitor. J Nanopart. Res. 16 2181(2014).

[b26] Wang L., Dong Z. H., Wang Z. G., Zhang F. X. & Jin J. Layered α-Co(OH)_2_ nanocones as electrode materials for pseudocapacitors: understanding the effect of interlayer space on electrochemical activity, Advanced Functional Materials. 23 2758–2764(2013).

