# Supplementary material for: Enhancing capacitance behaviour of CoOOH nanostructures using transition metal dopants by ambient oxidation
Source: Sci Rep. 2016 Feb 8;6:20704. doi: 10.1038/srep20704 (PMC4745070; doi:10.1038/srep20704)
Supplement: Supplementary Information [file srep20704-s1.pdf]

Supporting Information for

Enhancing capacitance behaviour of CoOOH nanostructures using transition metal dopants by ambient oxidation

Yanhui Chen[1,2*], Junfeng Zhou[2], Pierce Maguire[2], Robert O'Connell[2], Wolfgang Schmitt[3], Yonghe Li[1], Zhengguang Yan[1], Yuefei Zhang[1], Hongzhou Zhang[2]

[1] Institute of Microstructure and Property of Advanced Materials, Beijing University of Technology, Beijing, 100124, China
[2] School of Physics and Centre for Research on Adaptive Nanostructures and Nanodevices (CRANN), Trinity College Dublin, Dublin 2, Republic of Ireland
[3] School of Chemistry and Centre for Research on Adaptive Nanostructures and Nanodevices (CRANN), Trinity College Dublin, Dublin 2, Republic of Ireland
[*] Correspondence and requests for materials should be addressed to: yhchen@bjut.edu.cn

1 $Co_{0.9}Ni_{0.1}OOH$ nanorings formed at room temperature

1.1 EDX

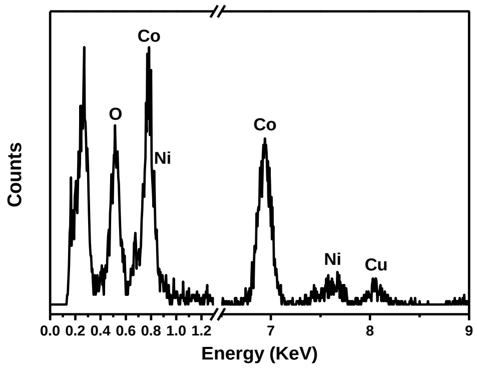

Fig. S1 EDX spectrum of typical nanorings.

1.2 Evolution of morphology

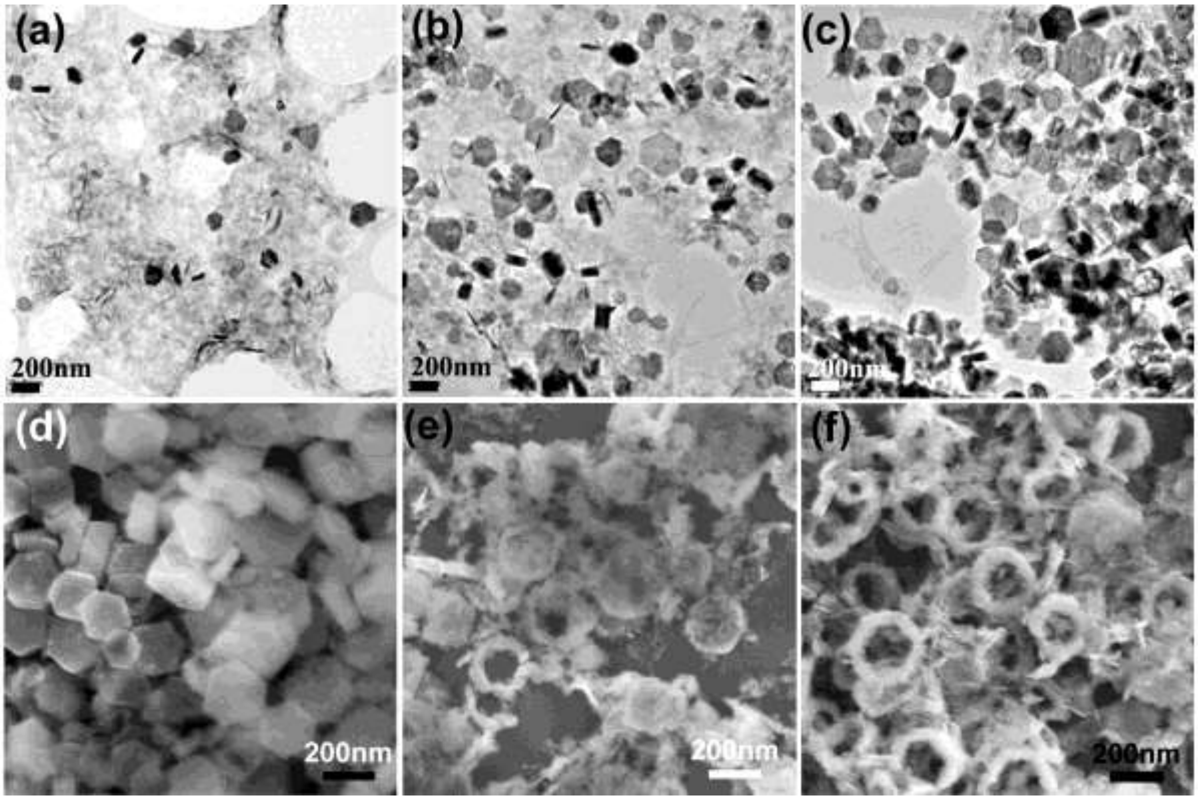

Fig. S2 TEM and SEM images showing the evolution of morphology after (a) 1 min, (b) 30 min, (c) 3 h, (d) 3 h, (e) 24 h and (f) 72 h.

Table S1 Ration of Ni in the nanodiscs in different reaction times.

Reaction time	Kind of the products	Ratio of Ni/(Ni+Co) (at%)
1 min	Nanodics	0%
	Gel-like products	10%
30 min	Nanodics	0%
	Gel-like products	10%
3 h	Nanodics	10-13%
	Gel-like products	Nearly dispear

1.3 SEM images on the effect of oxygen environment

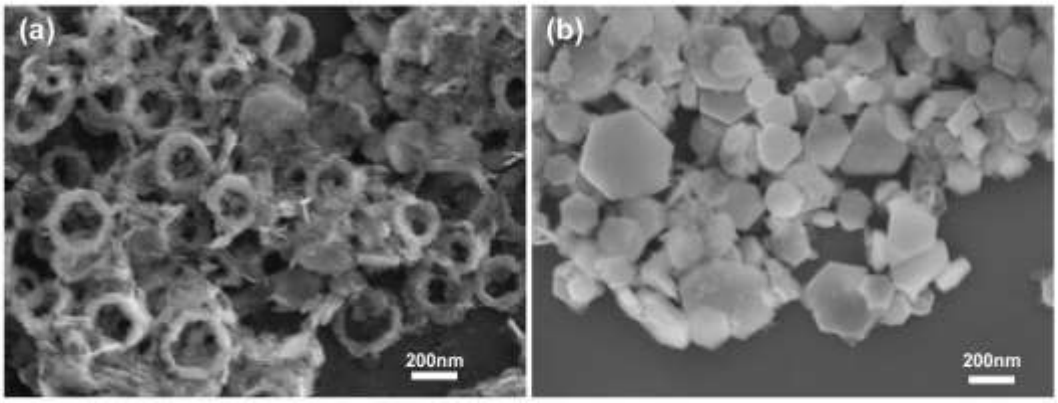

Fig. S3 (a) Nanorings formed by exposing to ambient and (b)nanodiscs formed using same concentration in a sealed tube.

1.4 SEM images on the effect of reductant $N_2H_4 \cdot H_2O$

Fig. S4 gives typical images of products synthesised using different amounts of $N_2H_4 \cdot H_2O$ in room temperature for 72 hours in open tubes. With the increase in the amount of $N_2H_4 \cdot H_2O$ from 0 to 25 µl, the products change from irregular plates of less than 50 nm (Fig. S4a) to 200-300 nm nanorings (Fig. S4b). The shapes then changed to 200-300 nm nanodiscs (Fig. S4c) When the $N_2H_4 \cdot H_2O$ was increased to 75 µl.

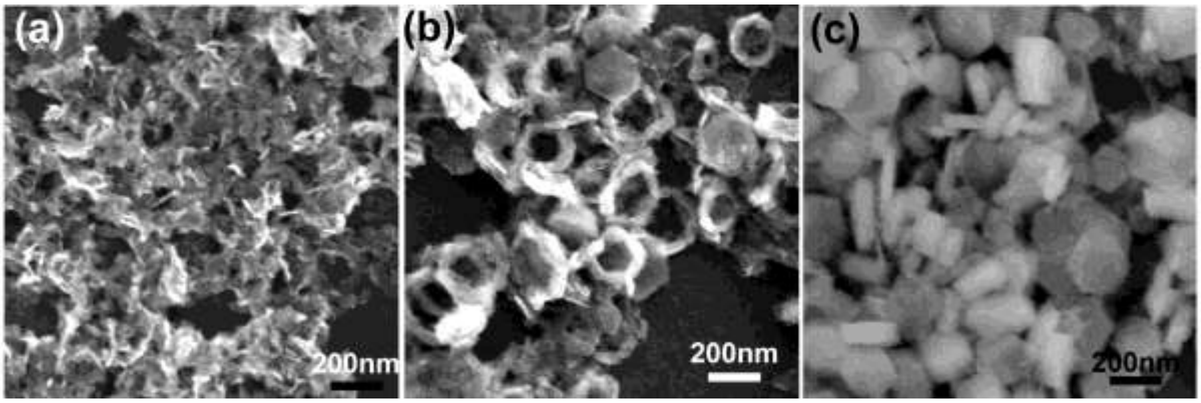

Fig. S4 SEM image of products by using different amount of $N_2H_4 \cdot H_2O$ (NaOH: 1 M, 20 ^oC/72 h) (a) 0 µl, (b) 25 µl and (c) 75µl.

1.5 SEM images on the effect of NaOH

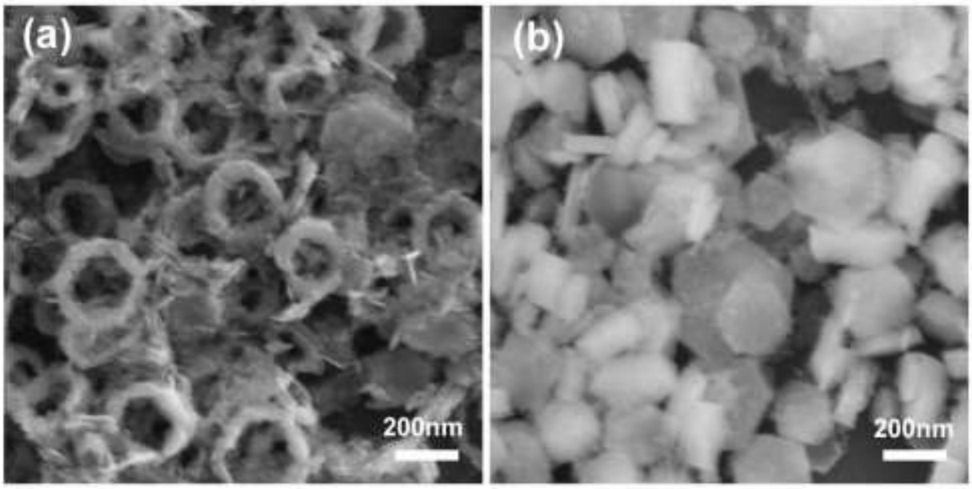

Fig. S5 SEM image of products by using different amount of N_aOH (N2H4: 25 µl) (a) 1 M, (b) 2 M.

2 CoOOH system

2.1 XRD and EDX

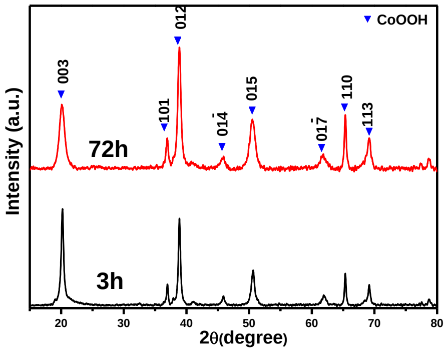

Fig. S6 XRD patterns of products after reaction for 3 h and 72 h.

2.2 SEM

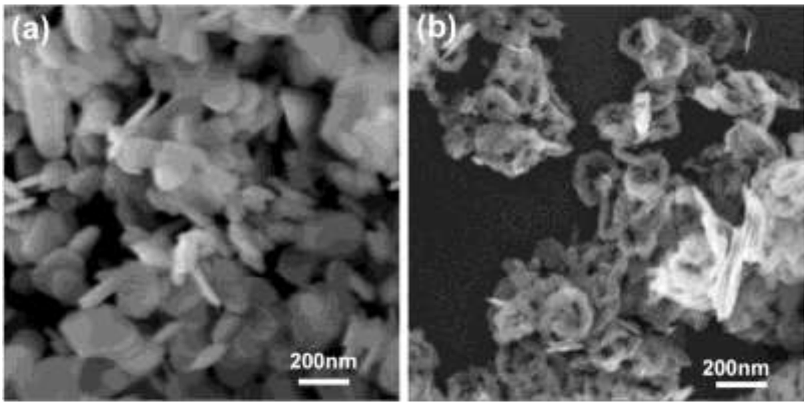

Fig. S7 SEM image of cobalt hydrate for reaction times of (a) 3 h and (b) 72 h.

2.3 Electrochemical properties

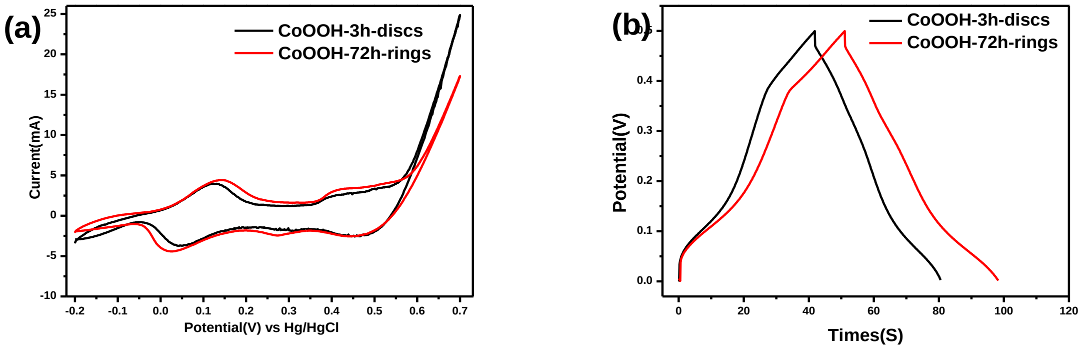

Fig. S8 (a) CV curves of CoOOH nanodiscs and CoOOH nanorings at a sweep rate of $5mVs^{-1}$ with a potential window of -0.2 to 0.7 V vs standard calomel electrode (SCE). (b) Galvanostatic charge/discharge curves of nanodiscs and nanorings at current densities of 1 Ag^{-1}.

3 $Co_{0.9}Mn_{0.1}OOH$ system

3.1 XRD and EDX

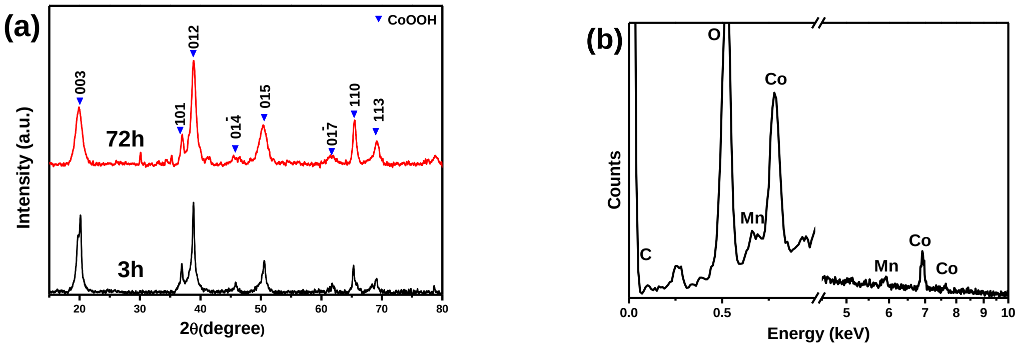

Fig. S9 (a) XRD patterns of products after reaction for 3 h and 72 h. (b) EDX spectrum of typical nanorings.

3.2 SEM

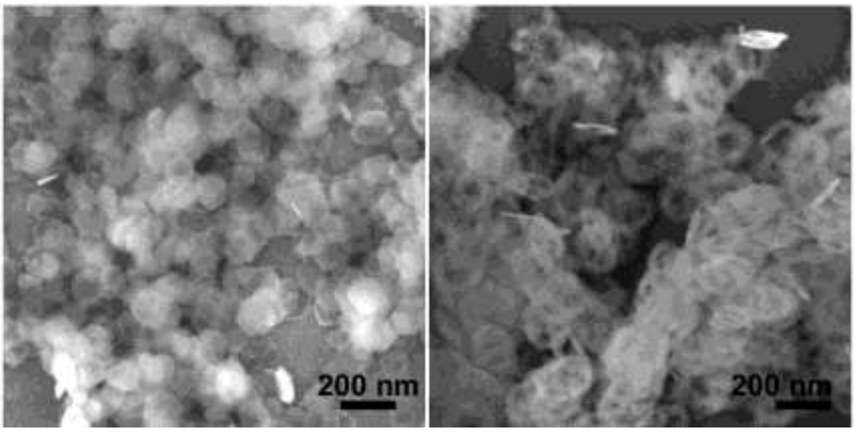

Fig. S10 SEM image of Mn doped cobalt hydrate for reaction times of (a) 3 h and (b) 72 h.

3.3 Electrochemical properties

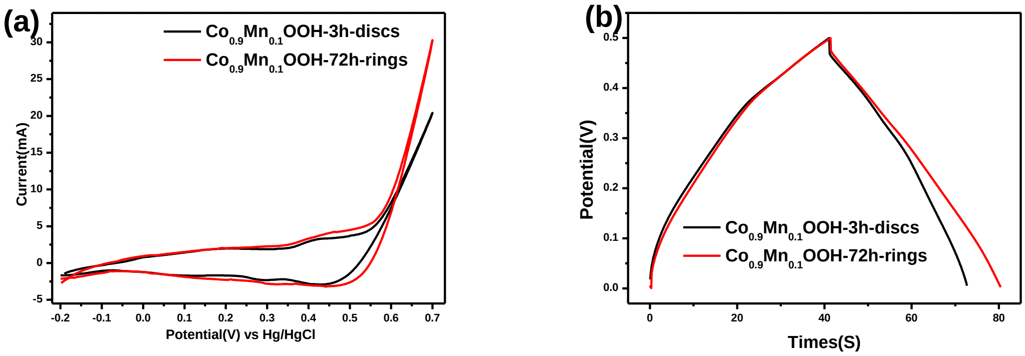

Fig. S11 (a) CV curves of $Co_{0.9}Mn_{0.1}OOH$ nanodiscs and nanorings at a sweep rate of 5 mVs^{-1} with a potential window of -0.2 to 0.7 V vs standard calomel electrode (SCE). (b) Galvanostatic charge/discharge curves of nanodiscs and nanorings at current densities of 1 Ag^{-1}.

4 $Co_{0.9}Fe_{0.1}OOH$ system

4.1 XRD and EDX

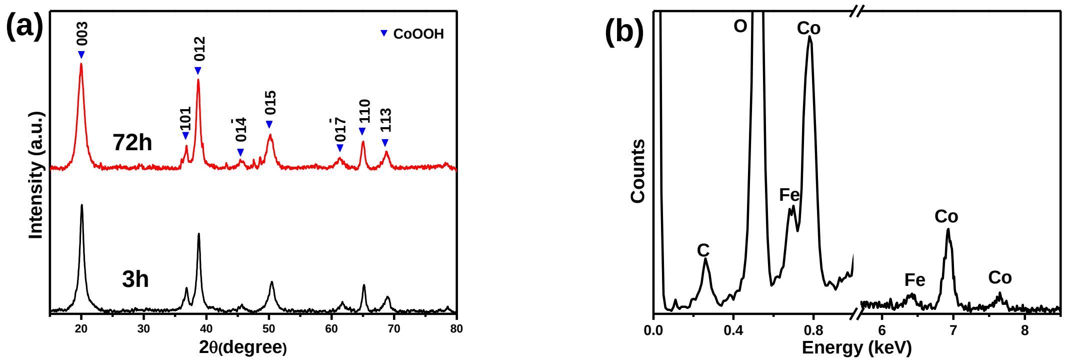

Fig. S12 (a) XRD patterns of products after reaction for 3 h and 72 h. (b) EDX of nanorings.

4.2 SEM

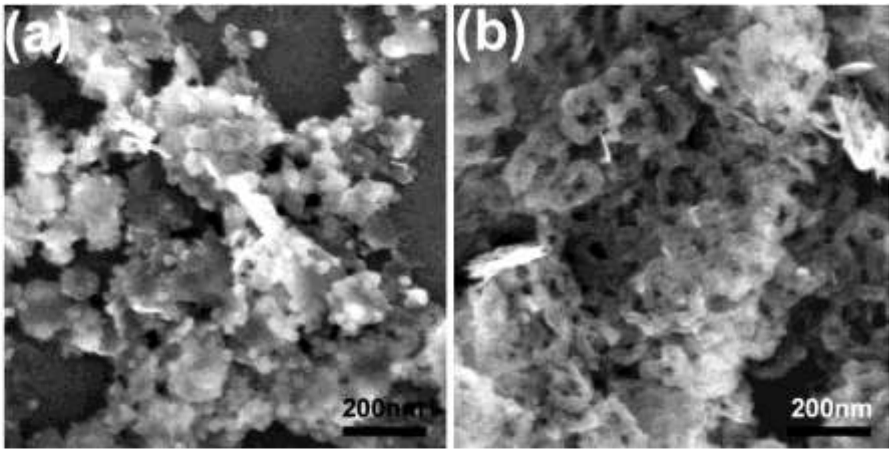

Fig. S13 SEM image of Fe doped cobalt hydrate for reaction times of (a) 3 h and (b) 72 h.

4.3 Electrochemical properties

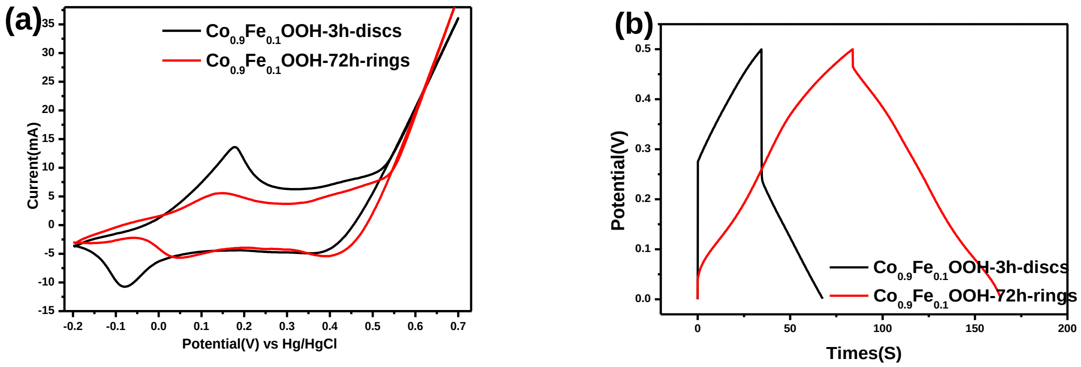

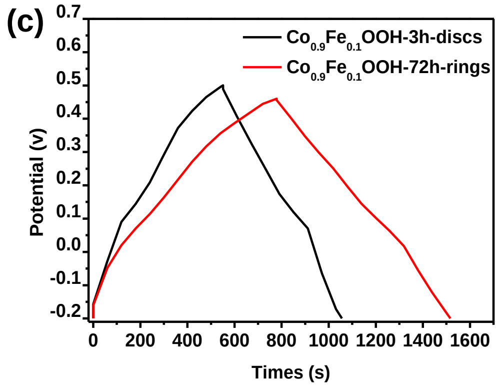

Fig. S14 CV curves of $Co_{0.9}Fe_{0.1}OOH$ nanodiscs and nanorings at a sweep rate of $5mVs^{-1}$ with a potential window of -0.2 to 0.7 V vs standard calomel electrode (SCE). (b) Galvanostatic charge/discharge curves of nanodiscs and nanorings at current densities of 1 Ag^{-1}. (c) Galvanostatic charge/discharge curves of nanodiscs and nanorings at current densities of 0.1 Ag^{-1}.
